# Calpain‐mediated cleavage of p53 in human cytomegalovirus‐infected lung fibroblasts

**DOI:** 10.1096/fba.1028

**Published:** 2018-12-03

**Authors:** Zhenping Chen, Paul J. Boor, Celeste C. Finnerty, David N. Herndon, Thomas Albrecht

**Affiliations:** ^1^ Department of Microbiology and Immunology University of Texas Medical Branch Galveston Texas; ^2^ Department of Pathology University of Texas Medical Branch Galveston Texas; ^3^ Department of Surgery University of Texas Medical Branch Galveston Texas; ^4^ Shriners Hospitals for Children^®^—Galveston Galveston Texas; ^5^ Infectious Disease and Toxicology Optical Imaging Core University of Texas Medical Branch Galveston Texas

**Keywords:** calpain, endogenous N‐terminal p53 fragments, human cytomegalovirus, p53, proteasome

## Abstract

Endogenous fragments of p53 protein were identified in human cytomegalovirus (HCMV)‐infected human lung fibroblasts, particularly a 44‐kDa N‐terminal fragment [hereafter referred to as p53(ΔCp44)], generated via calpain cleavage. The fragment abundance increased in a biphasic manner, peaking at 6‐9 hours and 48 hours post infection. Treatment of LU cells with calpain inhibitors eliminated most detectable p53 fragments. In cell‐free experiments, exogenous m‐calpain cleavage generated p53(ΔCp44). Attempts to preserve p53 proteins by treating cells with the calpain inhibitor E64d for 6 hours before harvesting increased the sensitivity of p53 to calpain cleavage. p53 in mock‐infected cell lysates was much more sensitive to cleavage and degradation by exogenous calpain than that in HCMV‐infected cells. The proteasome inhibitor MG132 stabilized p53(ΔCp44), particularly in mock‐infected cells. p53(ΔCp44) appeared to be tightly associated with a chromatin‐rich fraction. The abundance of p53β was unchanged over a 96‐h time course and very similar in mock‐ and HCMV‐infected cells, making it unlikely that p53(ΔCp44) was p53β. The biological activities of this and other fragments lacking C‐terminal sequences are unknown, but deserve further investigation, given the association of p53(ΔCp44) with the chromatin‐rich (or buffer C insoluble) fraction in HCMV‐infected cells.

**Abbreviations**
CTD: C‐terminal domain
DBD: DNA‐binding domain
HCMV: human cytomegalovirus
HTS: hypertrophic scar
LU cells: lung fibroblasts
NBS: non‐burned skin
OD: oligomerization domain
PCS: human primary dermal fibroblasts from normal neonatal foreskin
PFU: plaque‐forming unit
PI: post infection
PR: pro‐rich domain
PT: post treatment
TAD: transcription activation domain. GAPDH, glyceraldehyde 3‐phosphate dehydrogenase
αSMA: alpha smooth muscle actin

## 1 INTRODUCTION

The p53 tumor suppressor has been the subject of intense research over the last several decades. It is a key regulatory protein, especially as a transcription factor, and participates in diverse cellular processes, such as cell cycle arrest, DNA repair, and apoptosis.^1^, ^2^, ^3^, ^4^ p53 is also a translational regulator.^5^ Activities of p53, such as efficient and specific binding to p53 *cis*‐elements within target promoter sequences, as well as tissue‐, time‐, and stimulus‐specific binding of numerous coactivators and modifiers, are regulated by its abundance, post‐translational modifications, and protein‐protein interactions; all of which are influenced by a number of signaling pathways converging on p53.^1^, ^6^, ^7^, ^8^, ^9^, ^10^, ^11^, ^12^, ^13^, ^14^ Constitutive synthesis and degradation maintain low levels of p53 in unstressed cells, but provide a mechanism for the rapid increase in cellular p53 levels in response to stress through inhibition of p53 degradation.^4^, ^15^

Calpains are Ca^2+^‐activated cysteine proteases that participate in numerous physiological and pathological processes. They act through either general degradation or limited proteolysis, the latter of which allows them to modulate the functions of, rather than destroy, their substrates.^16^, ^17^, ^18^, ^19^, ^20^ It has been known for some time that human p53 may be degraded or cleaved by calpain^21^, ^22^, ^23^, ^24^, ^25^, ^26^ and that degradation of p53 by a calpain‐like protease is necessary for G_1_‐to‐S–phase transition.^21^ Cell‐free cleavage of p53 produced by in vitro translation in a rabbit reticulocyte lysate by m‐calpain results in the cleavage of p53 and generation of some sub‐53–kDa fragments.^23^ Some functions of p53 isoforms have been reported.^27^, ^28^, ^29^, ^30^, ^31^, ^32^, ^33^, ^34^, ^35^ For example, isoforms p53α, p53β, p53γ, Δ40p53α, and Δ133p53α have been shown to differentially regulate gene expression and to be biochemically and biologically active either alone or in combination with other isoforms.^32^ Furthermore, C‐terminal p53 isoforms that lack some amino acids in the N‐terminus may be associated with induction of p53 protein aggregation, which suppresses the normal functions of p53.^35^

Human cytomegalovirus (HCMV) is a β herpesvirus that is responsible for serious infections in the developing fetus and in individuals with compromised immunity.^36^ Replication of HCMV in quiescent host cells is dependent on activation of these cells to enter and traverse the cell cycle to a point at or near the G_1_/S boundary.^37^, ^38^, ^39^ Paradoxically, contrary to the low levels of p53 anticipated in cells stimulated to enter the cell cycle, p53 levels are substantially increased during productive HCMV infection^40^, ^41^, ^42^, ^43^, ^44^ and remain elevated for a protracted time during HCMV replication.^44^ In addition, our previous studies have shown that HCMV infection induces Ca^2+^ entry into infected cells,^37^ a substantial rise in intracellular free [Ca^2+^]^37^ and activation of the ubiquitous cellular calpains.^45^ µ‐ and m‐calpain activation temporally overlap the increase in cellular p53 levels.^45^ Thus, in HCMV‐infected cells, at the times when calpain activities are apparent,^45^ high cellular levels of p53 are available without the potential confounding effects of rapid ubiquitin‐facilitated p53 degradation.^44^

Endogenous p53 fragments generated by calpain‐mediated proteolytic cleavage in human cells have not been described. In this study, we conducted immunoblot analyses of HCMV‐infected cells and cell‐free calpain cleavage assays to investigate p53 cleavage and degradation in human lung fibroblasts (LU cells) during HCMV infection. The results indicate that endogenous fragments of p53, particularly a ~44‐kDa N‐terminal fragment [hereafter referred to as p53(ΔCp44)], were detected in HCMV‐infected LU cells, and m‐calpain‐mediated cell‐free cleavage of p53 yielded detectable levels of p53(ΔCp44). Subcellular localization analysis revealed that the fragment is tightly associated with the chromatin‐rich fraction.

## 2 MATERALS AND METHODS

### 2.1 Cell culture and growth arrest

Human LU cells^46^ were propagated in Eagle's minimum essential medium (Gibco, Life Technologies Corporation, Grand Island, NY) containing 10% fetal bovine serum (Intergen Co., Purchase, NY) and penicillin (100 units/mL)/streptomycin (100 µg/mL) (Gibco) at 37°C in 5% CO_2_. Human primary dermal fibroblasts HTS (obtained from hypertrophic scar tissue from a burn patient)^47^ and NBS (obtained from non‐burn skin tissue from the same patient)^47^; commercially available human primary dermal fibroblasts (PCS) were obtained from normal neonatal foreskin (ATCC® PCS‐201‐010™). The dermal fibroblasts were cultured in Dulbecco's Modified Eagle's Medium (Corning Life Sciences, Manassas, VA, Catalog # 10‐013‐CV) supplemented with 15% fetal calf serum (Gibco, Catalog # 10437028), and 1% antibiotic‐antimycotic (100x solution, Gibco, Catalog # 15240062). The isolation of two human primary dermal fibroblast cell lines (HTS, NBS) and the use of the four human fibroblast cell lines were approved by the Institutional Review Board at the University of Texas Medical Branch, Galveston, TX.

### 2.2 Virus stocks and productive infection

The AD169 strain of HCMV was propagated in LU cells as previously described.^48^ The infectivity of virus stocks was determined by plaque assay.^49^ Virus stocks typically had infectivities between 8.0 × 10^6^ and 4.0 × 10^7^ PFU/mL. The cells were density‐arrested as detailed previously,^50^ then infected with HCMV at a multiplicity of 5 PFU/cell to provide a uniform infection as described previously.^48^ Virus stocks and cell cultures were routinely examined for mycoplasma and were free from detectable contamination.

### 2.3 Chemicals

The calpain inhibitors E64d (Product Code: IED‐4321‐v) and Z‐Leu‐Leu‐H (ZLLH, Product Code: IZL‐3178‐v) were purchased from Peptides International, Inc (Louisville, KY). Human µ‐Calpain (calpain II, catalog # C6108), penicillin, streptomycin, Tris, NaCl, NaVO_3_, NaF, phenylmethylsulfonyl fluoride (PMSF), dithiothreitol (DTT), trypsin inhibitor, aprotinin, benzamide, and pepstatin A were purchased from Sigma‐Aldrich Corp. (St. Louis, MO). Human m‐Calpain (calpain I, catalog # 208713) and NP‐40 were obtained from EMD Millipore Corporation (Bedford, MA).

### 2.4 Immunoblots

Whole‐cell extracts (40 µg/lane) were fractionated on 4%‐12% NuPAGE^TM^ SDS‐polyacrylamide gels (NP0321, Invitrogen Life Technologies Corporation, Grand Island, NY). Amersham™ ECL™ Rainbow™ Marker (RPN800E, GE Healthcare Bio‐Sciences, Pittsburgh, PA) was loaded in a lane in each gel and separated with the protein samples simultaneously as a reference for estimating the molecular weights of protein bands. The polypeptides were transferred to polyvinylidene difluoride (PVDF) membranes (Bio‐Rad Laboratories, Hercules, CA), as previously described.^45^, ^51^ Antibodies specific for p53, namely DO‐1 (sc‐126), Bp53‐12 (sc‐263), Pab240 (sc‐99), C‐19 (sc‐1311‐R), and FL393 (sc‐6243), and antibodies against actin (sc‐7210), PCNA (sc‐56), and α‐tubulin (sc‐5546) were obtained from Santa Cruz Biotechnology, Inc (Dallas, TX). Antibody against calpain was a gift of Dr. R. L. Mellgren.^52^ The antibody specific for p53β (KJC8) was a gift from Drs. J. C. Bourdon and D. P. Lane.^28^ The antibody specific for HCMV IE1 protein was a gift of Dr. E. S. Huang.^53^ Antigen‐antibody reactions were detected with the enhanced chemiluminescent assay (Amersham Pharmacia Biotech, Piscataway, NJ) following the manufacturer's recommendations. Immunoblots were exposed to Biomax XAR film (Kodak, Rochester, NY).

### 2.5 Cell fractionation and protein extraction

The methods of Lo et al^54^ were used with some modifications (described below) to separate nuclear and cytoplasmic fractions. Cells in 100‐mm tissue culture dishes were washed with Dulbecco's PBS and scraped off the plastic surface with a cell lifter. Preliminary experiments revealed that the cleavage fragments were not stable, whereas treating the cells with MG132 could stabilize the fragments. Thus, MG132 (10 µM) was added to the lysis buffer during the protein extraction for all subsequent experiments (ie, immunoblotting and calpain cleavage assays). The cells were collected by sedimentation. To obtain the cytoplasmic fraction, the cells were resuspended in hypotonic buffer [5 mM KCl, 0.5 mM DTT, 25 mM Tris‐HCl (pH 7.5), 1 mM PMSF, 25 µg/mL aprotinin, 10 µM MG132] and allowed to swell for 20 minutes on ice. All subsequent steps were also performed on ice. The swollen cells were placed in a chilled Dounce homogenizer, and the cytoplasmic membranes were disrupted. The nuclei were collected by sedimentation (600× *g* for 10 minutes at 4°C). The cytosolic lysates (supernatants) were transferred to fresh centrifuge tubes. To prepare nuclear extracts, the collected nuclei (pellets) were washed three times in isotonic buffer [0.25 M sucrose, 6 mM MgCl_2_, 10 mM Tris‐HCl (pH 7.4), 0.1% Triton X‐100, 1 mM PMSF, 25 µg/mL aprotinin, 10 µM MG132], and the final nuclear suspension was examined under a microscope for the presence of contaminating cellular debris. (a) To extract the nuclear proteins for calpain cleavage assays, nuclei were resuspended in sonication buffer (25 mM Tris‐HCl [pH 7.5], 1 mM DTT, 0.1% Triton X‐100, 1 mM PMSF, 25 µg/mL aprotinin, 10 µM MG132, 1 mM NaVO_3_)^54^ and lysed using a Branson Cell Disruptor 185 probe sonicator. The nuclear lysate was clarified by sedimentation at 14,000 rpm for 20 minutes at 4°C. Protein extracts were stored in liquid nitrogen. (b) To extract the nuclear proteins for immunoblot, we resuspended nuclei in high‐salt extraction buffer (Buffer C)^55^ and incubated them on ice for 20 minutes. The nuclear lysate was clarified by centrifugation at 14,000 rpm for 20 minutes at 4°C. The supernatants, which contained Buffer C‐soluble nucleoplasm, were collected and used for immunoblot analysis. Buffer C contains 20 mM HEPES‐KOH (pH 7.9), 25% Glycerol, 420 mM NaCl, 1.5 mM MgCl_2_, 0.2 mM EDTA, 0.5 mM DTT, 0.2 mM PMSF, and MG132 (10 µM). The pellets, which contained the Buffer C‐insoluble nuclear proteins, were then extracted using the SUMO‐1–modified protein extraction buffer,^56^ which consisted of a 1:3 mixture of buffer I [5% SDS, 0.15 M Tris‐HCl (pH 6.8)], 30% glycerol] and buffer II [25 mM Tris‐HCl (pH 8.3), 50 mM NaCl, 0.5% NP‐40, 0.5% deoxycholate, 0.1% SDS], supplemented with protease inhibitors (25 µg/mL aprotinin, 1 mM PMSF, 25 µg/mL trypsin inhibitor). When the Buffer C‐insoluble pellets were carefully resuspended, they became highly viscous, so the samples were briefly sonicated to fragment the DNA^57^ and to reduce sample viscosity. They were then centrifuged at 14,000 rpm at 4°C for 15 minutes. The resulting supernatants containing the Buffer C‐insoluble fraction were used for immunoblot analysis.

### 2.6 Cell‐free digestion of p53 with purified calpain

Digestion was performed with 160 µg of nuclear protein extract. All samples were kept on ice until digestion was initiated. The volume of each sample was adjusted to 18 µL by adding an appropriate volume of sonication buffer.^54^ Each sample was then mixed with 20 µL of 2× cleavage buffer to bring the volume to 38 µL and with 2 µL of purified recombinant human calpain, containing 0.08, 0.04, 0.02, 0.01, or 0.00 units of either µ‐ or m‐calpain. The calpain solutions were prepared by series dilution in 1× cleavage buffer just before initiation of the digestion assays. In each test series, one of the two control samples received 2 µL of 200 mM EDTA, instead of 2 µL of 0.00 unit calpain, to block endogenous Ca^2+^‐dependent proteases, including calpain, before digestion. This control sample served as a reference control for cleavage by endogenous calpain and other Ca^2+^‐dependent proteases. The final volume was 40 µL in 1× cleavage buffer, which contained 25 mM Tris‐HCl (pH 7.5), 100 mM NaCl, 3 mM DTT, and 5 mM CaCl_2_. The digestion was started as soon as possible by incubating the mixture at 30°C for 10 minutes. The samples were then transferred to ice, and the digestion was terminated by adding 2 µL of 200 mM EDTA to the reaction mixture. For the control sample to which EDTA had already been added, 2 µL sonication buffer was added instead of ETDA after digestion. The 4× sample buffer (NP0007, Invitrogen) was then mixed 1:3 with the digestion mixture and incubated at 70°C for 10 minutes to denature the proteins. Each digested sample/lane (80 µg) was then evaluated by immunoblot analysis.

## 3 RESULTS

### 3.1 Identification of p53‐related polypeptides of less than 53 kDa in HCMV‐infected cells

**Figure 1:**
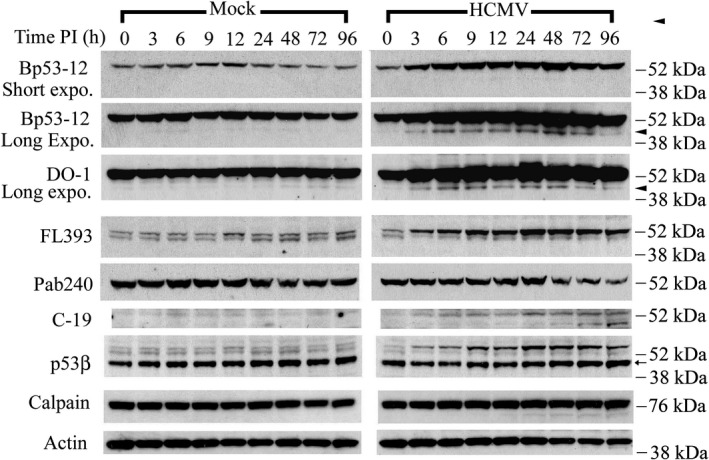
Identification of sub‐53–kDa polypeptides in HCMV‐infected cells, as revealed by a panel of p53 antibodies. Permissive human lung fibroblasts were density arrested and infected with HCMV (5 PFU/cell) or mock infected. Whole‐cell lysates were prepared at the indicated times post infection (PI). Protein aliquots (40 μg/lane) were resolved and membranes probed with the indicated p53 antibodies. Membranes were reprobed with antibody to m‐calpain or actin. These results are representative of at least two independent biological replicates, each one in two technical replicates. Expo: exposure. Arrowhead: p53(ΔCp44). Arrow: p53β. kDa sizes: the relevant molecular weight markers

The fate of p53 in HCMV‐ and mock‐infected LU cells was investigated using whole‐cell lysates and a panel of antibodies to p53 (Table 1). Analysis of protein blots using the p53 antibodies Bp53‐12^58^ and DO‐1^58^ revealed that, in mock‐infected LU cells, full‐length p53 was present at a relatively low level throughout the 96‐hour course of these experiments (Figure 1). In contrast, p53 abundance increased in HCMV‐infected cells as the infection progressed. Similar increases in p53 following HCMV infection have been reported by our group^44^ based on immunoblot analyses using DO‐1 (Table 1) and by others^40^, ^41^, ^42^ based on immunoblot analyses using the antibodies DO‐1, DO‐7 and Bp53‐12. These antibodies target the p53 N‐terminus.^58^ A similar increase in p53 abundance was observed when the antibody FL393 was used for immunoblot analysis (Figure 1). Although immunoblot analysis with C‐19 also revealed a substantial increase in p53 in HCMV‐infected cells, the levels were not as great as those observed with the former antibody preparations and a biphasic increase was seen with maxima at 3‐9 hours post infection (PI; peak at 6 hours) and 24‐96 hours PI (peak at 72 hours). Paradoxically, immunoblots incubated with the antibody Pab240, which targets p53 amino acids 156 to 214 (Table 1), showed a decrease in p53 at 48‐96 hours PI. Thus, p53 levels appeared to increase substantially with HCMV infection when antibodies decorating the N‐terminal sequences or the full‐length molecule were used. However, smaller increases were seen with an antibody reactive with C‐terminal sequences (C‐19) and decreases were observed with an antibody targeting an epitope near the middle of the molecule (Pab240).

**Table 1:** Reactivity of antibodies to p53 epitopes and fragment sizes

Antibody	Targeted aa	Fragment (kDa)
DO‐1	10 to 15	~44 (~47 and ~50)
Bp53‐12	N′‐terminus	~44 (~47 and ~50)
Pab240	156 to 214	Undetected
FL393	1 to 393	~50
C‐19	C'‐terminus	~47
KJC8	Intron 9 of p53β	46

aa, amino acids.

To better characterize less abundant p53 fragments, immunoblots were exposed to films for a longer time. Using all antibodies described above (Table 1) except Pab240, lower molecular mass bands that were rather faint were consistently observed (Figure 1). Bp53‐12 and DO‐1 detected a ~ 44‐kDa p53 N’‐terminal fragment, p53(ΔCp44), that showed a biphasic increase, peaking at 6‐9 hours and 48 hours after HCMV infection. A sub‐p53 fragment was not detected during mock‐infection at the same film exposure time. These results suggest that, in addition to increasing full‐length p53, HCMV infection substantially increased p53(ΔCp44). FL393 consistently revealed a band of about 50‐kDa in extracts of mock‐ and HCMV‐infected cells; however, p53(ΔCp44) was not observed with FL393 (Figure 1). Unlike the 53‐kDa polypeptide, the ~50‐kDa fragment detected with FL393 did not appear to increase over the duration of these experiments. C‐19 detected a faint band with an approximate mass of 45‐kDa that was not consistently detected during the early phase of HCMV infection. This band became more intense as the infection progressed (particularly at 72 and 96 hours PI), ultimately having an intensity similar to full‐length p53 by 96 hours PI. p53(ΔCp44) was not detected with C‐19 in either mock‐ or HCMV‐infected cells. Pab240 did not detect sub‐53–kDa polypeptides in either HCMV‐ or mock‐infected cells.

Twelve human p53 isoforms (p53α, p53β, p53γ, ∆40p53α, ∆40p53β, ∆40p53γ, ∆133p53α, ∆133p53β, ∆133p53γ, ∆160p53α, ∆160p53β, and ∆160p53γ) resulting from alternative transcription initiation and splicing sites have been reported.^27^, ^28^, ^29^, ^30^, ^31^, ^32^ Moreover, an alternative translation initiation site has been identified for p53. This results in the formation of p53/47,^59^ also known as ΔNp53.^27^, ^60^ Because ∆133p53 and ∆160p53 isoforms, and p53/47 lack the N‐terminus that reacts with DO‐1^58^ and Bp53‐12^58^ (Table 1), the p53(ΔCp44) band detected in HCMV‐infected cells is unlikely to be these p53 isoforms. However, p53β, which corresponds to p53i9, arises from alternative splicing of intron 9 and is truncated, lacking the last 60 amino acids. Accordingly, p53β has a close molecular mass (46 kDa) and contains N‐terminal sequences that would be expected to react with Bp53‐12 and DO‐1. To investigate the possibility that p53(ΔCp44) corresponded to p53β, we probed blots with a p53β‐specific antibody raised against the intron 9 epitope, which is not present in wild‐type p53.^28^ The p53β‐specific antibody detected an approximate 46‐kDa band with an intensity that remained unchanged over the 96‐hour time course and was very similar in mock‐ and HCMV‐infected cells (in Figure 1). Therefore, the p53(ΔCp44) associated with HCMV infection that was detected using Bp53‐12 or DO‐1 did not appear to be p53β. Interestingly, two more slowly migrating species larger than 53 kDa were observed with the p53β antibody in the HCMV‐infected cells, with the heavier one increasing in intensity during the virus infection. Whether this band possessing the intron 9 epitope may represent a new isoform remains to be investigated. Since an antibody specific for p53γ was not available, its expression in HCMV‐infected LU cells also remains to be investigated.

While the increase in p53(ΔCp44) detected with Bp53‐12 and DO‐1 appeared to be specifically associated with HCMV infection, levels of the FL393‐reactive ~50‐kDa species only slightly changed during HCMV infection. Moreover, this ~50‐kDa band was also detected in mock‐infected cells with rather consistent intensity throughout the 96‐hour time course. In addition, the ~45‐kDa C‐terminal species was not detected consistently with C‐19 antibody. For these reasons, subsequent experiments focused on p53(ΔCp44).

### 3.2 Calpain inhibitors substantially reduce levels of p53(ΔCp44)

**Figure 2:**
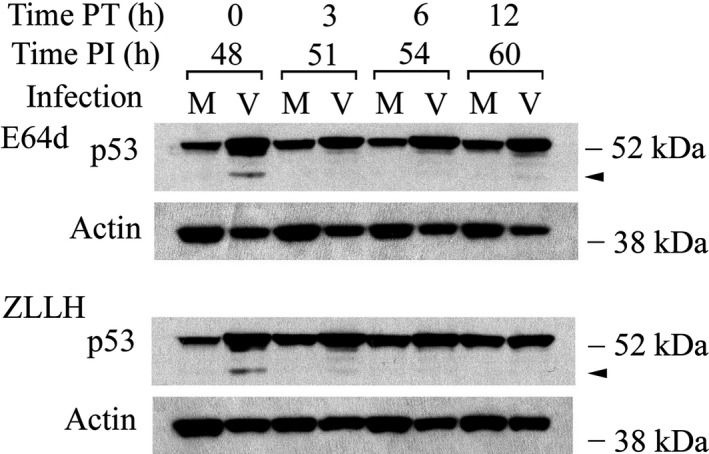
Calpain inhibitors eliminate most p53(ΔCp44) in HCMV‐infected cells. Cells were treated with calpain inhibitors [E64d (100 µM) or ZLLH (100 µM)] at 48 h PI and harvested at the indicated times post treatment (PT). Polypeptides (40 μg/lane) were resolved by SDS‐PAGE. Blots were probed with p53 antibody (DO‐1) and reprobed with actin antibody. These results are representative of at least two independent biological replicates, each one in two technical replicates. Note that the film exposure time for these DO‐1 immunoblots was shorter than those for DO‐1 immunoblots in Figure 1. M, mock‐infected; V, HCMV‐infected. Arrowhead: p53(ΔCp44). kDa sizes: the relevant molecular weight markers

If p53(ΔCp44) identified in lysates of HCMV‐infected cells was a product of the proteolytic activity of calpains, then treatment with calpain inhibitors should preserve the full‐length 53‐kDa protein and substantially reduce or eliminate p53(ΔCp44). Accordingly, density‐arrested LU cells were HCMV‐ or mock‐infected for 48 hours, treated with calpain inhibitors [E64d (100 µM) or ZLLH (100 µM)], and harvested at intervals from immediately after treatment to 12 hours post treatment (PT). Polypeptides extracted from cell lysates were separated by SDS‐PAGE, and blots were probed with DO‐1 (Figure 2). In mock‐infected cells, full‐length p53 increased after treatment with ZLLH. However, bands corresponding to the ~44‐kDa and ~47‐kDa fragments were not detectable. In HCMV‐infected cells, at the first time point (0 hour after E64d or ZLLH treatment), a broad upper band appearing to correspond to full‐length p53 was present. However, upon closer inspection, this band was found to be a doublet of 53‐kDa and ~50‐kDa bands. Below these bands, p53(ΔCp44) was evident, while the ~47‐kDa fragment band was very faint. After 3 to 12 hours of treatment with either E64d or ZLLH, the intensity of the 53‐kDa band detected by DO‐1 remained at about the same intensity as at 0 hour PT; while the ~44‐, ~47‐ and ~50 kDa fragment bands were substantially decreased or even undetectable by 3 hours PT. Thus, p53(ΔCp44) was sensitive to the calpain inhibitors E64d and ZLLH, suggesting that calpain activity was associated with the generation of this fragment.

### 3.3 MG132 stabilizes p53(ΔCp44)

**Figure 3:**
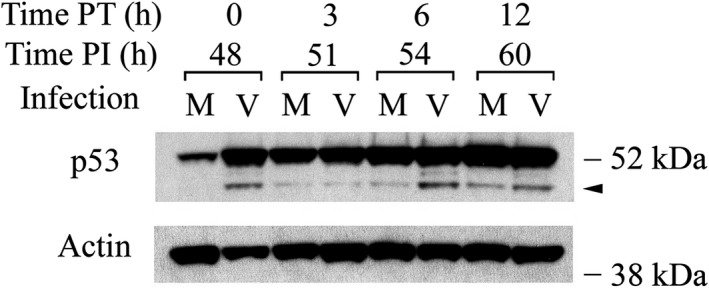
The proteasome inhibitor MG132 enhances the abundance of p53(ΔCp44). Cells were treated with or without MG132 (10 µM) at 48 hours PI and harvested at the indicated time post treatment (PT). Blots were probed with p53 antibody (DO‐1) and reprobed with actin antibody. These results are representative of at least two independent biological replicates, each one in two technical replicates. Note that the film exposure for these DO‐1 immunoblots was shorter than that for DO‐1 immunoblots in Figure 1. M, mock‐infected; V, HCMV‐infected. Arrowhead: p53(ΔCp44). kDa sizes: the relevant molecular weight markers

Many important regulatory proteins are degraded through the ubiquitin‐proteasome pathway.^61^ MG132 inhibits proteasome‐mediated degradation and consequently stabilizes proteins degraded through this pathway. In most experiments, the sub‐53–kDa bands were not detectable in mock‐infected cells. Possibly, these fragments were unstable. If they were degraded via the ubiquitin pathway, then blocking that pathway should preserve them and provide a more sensitive indication of the relative abundance of the sub‐53–kDa fragments in mock‐ and HCMV‐infected cells. Accordingly, mock‐ and HCMV‐infected cells were treated with MG132 (10 µM) at 48 hours PI, and the cells were harvested up to 12 hours PT. Indeed, p53(ΔCp44) was stabilized in both mock‐ and HCMV‐infected cells after MG132 treatment (Figure 3). We^44^ and others^62^ have described the stabilizing effect of MG132 on full‐length p53. In mock‐infected cells, MG132 treatment progressively increased p53(ΔCp44) abundance, with modest increases being seen at 3 hours PT and substantial increases being seen at 12 h PT. In HCMV‐infected cells, MG132 decreased the p53(ΔCp44) intensity at 3 hours PT, but increased it by 6 hours PT. At 12 hours PT, this increase had dwindled slightly, though levels remained higher than at 3 hours PT and higher than levels in mock‐infected cells at 12 PT. These results suggest that levels of p53(ΔCp44) are at least partially controlled by proteasome degradation.

**Figure 4:**
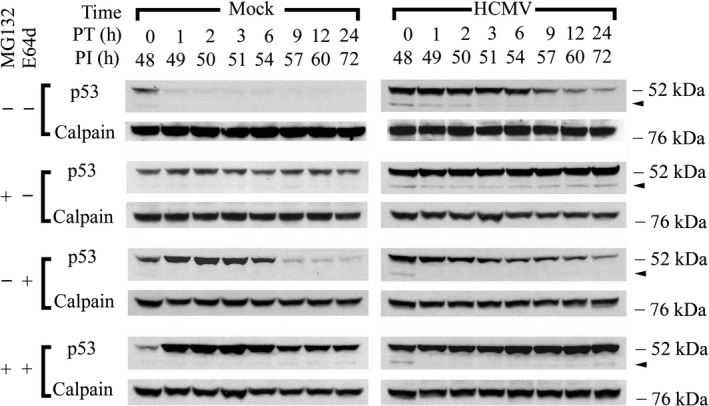
Effect of proteasome and/or calpain inhibition on the stability of p53(ΔCp44). Cells were treated with cycloheximide (100 µg/mL), with or without either the proteasome inhibitor MG132 (10 µM) and/or the calpain inhibitor E64d (100 µM) at 48 hours PI and harvested at the indicated time post treatment (PT). Blots were probed with p53 antibody (DO‐1) and reprobed with m‐calpain antibody. These results are representative of at least two independent biological replicates, each one in two technical replicates. Arrowhead: p53(ΔCp44). kDa sizes: the relevant molecular weight markers

To investigate further if p53(ΔCp44) was degraded via the ubiquitin‐proteasome pathway, as well as the possible interplay between the ubiquitin‐proteasome pathway and any calpain‐mediated degradation of p53(ΔCp44), the stability of p53(ΔCp44) after inhibiting the proteasome and calpain pathways either separately or in combination was evaluated. Forty‐eight hours after mock‐ or HCMV‐infection, protein synthesis was blocked with cycloheximide (100 µg/mL), and the cells were either treated with E64d (100 µM) and/or MG132 (10 µM) or left untreated (control). Levels of p53 and p53(ΔCp44) were then followed over time by immunoblot (Figure 4). In mock‐infected cells treated with only cycloheximide, full‐length p53 had a very short half‐life (<1 hour), as we^44^ and others^4^ have previously shown. p53(ΔCp44) was detectable as a very faint band at 0 hour PT and thereafter was undetectable (Figure 4, left column, top row). In HCMV‐infected cells treated with cycloheximide, full‐length p53 had a much longer half‐life (about 9 hours), as we have previously observed,^44^ while the half‐life of p53(ΔCp44) was extended by only an hour (Figure 4, right column, top row) relative to that in mock‐infected cells at 0 hour PT. After MG132 treatment of mock‐ and HCMV‐infected cells, levels of full‐length p53 remained relatively constant or slightly decreased over the 24 hours time course, respectively. Although only a very faint hint of p53(ΔCp44) could be seen in mock‐infected cells, this fragment was visible at all of the time points in HCMV‐infected cells and the half‐life was substantially extended to over 24 hours (Figure 4, second row). In mock‐infected cells, E64d extended the half‐life of full‐length p53 to about 6 hours, but did not enhance the half‐life of p53(ΔCp44) (Figure 4, left column, third row). In HCMV‐infected cells, E64d did not stabilize either full‐length p53, or p53(ΔCp44). In fact, the intensity of the p53(ΔCp44) band was diminished in HCMV‐infected cells (Figure 4, right column, third row). Co‐incubation with E64d and MG132 increased the abundance of full‐length p53 and p53 fragments in mock‐infected cells relative to treatment with either MG132 or E64d alone, particularly at 1 to 6 hours PT. Similar to mock‐infected cells treated with MG132 alone, those co‐treated with E64d and MG132 revealed full‐length p53 with half‐lives exceeding 24 hours (Figure 4, left column, bottom row). In HCMV‐infected cells, E64d, when co‐treated with MG132, seemed to have only a modest effect on increasing the abundance and the stability of full‐length p53 beyond that of MG132 treatment alone. On the other hand, when combined with MG132, E64d abolished the MG132‐induced enhancement of the half‐life of the p53 fragment (Figure 4, right column, bottom row). These results show further that most p53 protein and p53(ΔCp44) were degraded through the proteasome pathway in mock‐infected cells and to a lesser extent, in HCMV‐infected cells, in which the proteasome pathway is compromised.^44^ The ability of calpain inhibition to stabilize full‐length p53 in mock‐infected cells, without having much effect on p53 in HCMV‐infected cells, indicates that a large portion of p53 molecules were sensitive to calpain degradation in mock‐infected cells, and resistant to calpain degradation in HCMV‐infected cells. The finding that calpain inhibition substantially reduced abundance of p53(ΔCp44) in HCMV‐ and mock‐infected cells further support the view that calpain was involved in the generation of this fragment.

### 3.4 The sensitivity of p53 in mock‐ and HCMV‐infected cell lysates to cell‐free cleavage by exogenous calpain and generation of sub‐53–kDa fragments

**Figure 5:**
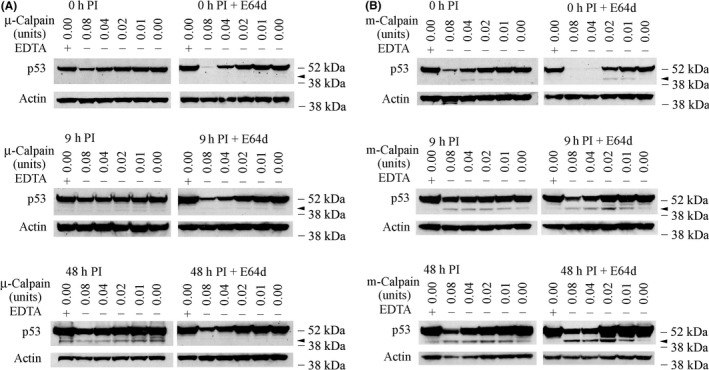
Sensitivity of p53 in HCMV‐infected cell lysates to cell‐free cleavage by purified µ‐ or m‐calpain and generation of p53(ΔCp44) by purified m‐calpain. HCMV‐infected cells were treated with or without 100 µM E64d 6 hours before harvest to help preserve calpain‐sensitive p53. Nuclear protein (160 µg) was isolated at the indicated time post infection (PI) and incubated with 0.08 units or less of purified µ‐calpain or m‐calpain for 10 minutes at 30°C. In one of the two control samples in each cleavage assay, calpain was replaced with EDTA, which inactivates endogenous Ca^2+^‐dependent proteases (lane 1). Protein extracts (80 µg) were then analyzed by immunoblot with DO‐1 and reprobed with actin antibody. The results are representative of at least two independent biological replicates, each one in two technical replicates. Arrowhead: p53(ΔCp44). kDa sizes: the relevant molecular weight markers

To evaluate whether HCMV infection is associated with possible conformational changes in the p53 protein that affect its susceptibility to calpain‐mediated cleavage and generation of the observed p53 fragments, we extracted nuclear proteins from mock‐ or HCMV‐infected cells and exposed them to exogenous purified human µ‐ or m‐calpain. Cleavage of p53 was assessed by immunoblot analysis using DO‐1 antibody. Nuclear proteins were selected for the cleavage assays because most p53 is located in the nuclei of HCMV‐infected cells.^44^ Furthermore, as HCMV infection progresses, a series of cellular physiologic responses occur.^37^, ^38^, ^39^ The availability of viral^63^, ^64^, ^65^, ^66^, ^67^, ^68^ and cellular molecules that can interact with p53 changes during the progression of HCMV infection, making it possible that p53 undergoes modifications that influence its susceptibility to calpain‐mediated cleavage. Considering these and other findings,^44^ we evaluated the sensitivity of p53 in nuclear extracts prepared at 0, 9, and 48 hours PI to exogenous calpain‐mediated degradation. Cleavage assays performed with exogenous µ‐calpain and extracts from 0 hour HCMV‐infected cells showed a concentration‐dependent reduction in full‐length p53 (Figure 5A, left column) (Note that the film exposure time was not as long as that for the film shown in Figure 1). However, sub‐53–kDa fragments detected with DO‐1 were not increased relative to fragments generated in assays without exogenous µ‐calpain (lane 6). In the absence of EDTA and exogenous µ‐calpain, multiple sub‐53–kDa fragments were more evident at 48 hours PI (lane 6, lowest panel) than at 0 or 9 hours PI (upper and middle panels). Moreover, incubation with the exogenous µ‐calpain did not generate a greater abundance of p53(ΔCp44) than that observed in the absence of exogenous µ‐calpain (compare the findings for lane 6 with those for lanes 2‐5), while addition of EDTA to inactivate endogenous Ca^2+^‐activated proteases during cleavage assays reduced the abundance of p53(ΔCp44) (lane 1). Thus, an endogenous Ca^2+^‐dependent protease(s), rather than exogenous µ‐calpain, may have cleaved p53 to generate the observed p53 fragments. At 48 hours PI, the p53 fragments appeared to be further degraded in a concentration‐dependent manner by exogenous µ‐calpain, as was full‐length p53, without increasing the abundance of p53(ΔCp44).

Possibly, only some p53 molecules were susceptible to µ‐calpain‐mediated cleavage in HCMV‐infected cells, and the p53 remaining in the lysates might largely represent calpain‐resistant p53 molecules. If this hypothesis were correct, then most of the p53 sensitive to µ‐calpain cleavage would have been cleaved in the HCMV‐infected cells before the in vitro cleavage assays were initiated. Accordingly, preserving calpain‐susceptible p53 molecules in the protein extracts could provide better insight into the degree to which sub‐53–kDa fragments could be generated by exogenous µ‐calpain. To test this possibility, we treated mock‐ and HCMV‐infected cells with 100 µM E64d for 6 hours before harvest to enhance preservation of calpain‐sensitive p53. At the time of harvest, the cells were carefully washed with PBS to remove the E64d before sample extraction. Using these samples in cell‐free µ‐calpain cleavage assays, we found that, indeed, at all times after infection, E64d‐protected p53 was much more sensitive to cleavage by exogenous µ‐calpain (Figure 5A, right column) than non‐protected p53 (Figure 5A, left column). Remarkable degradation of p53 was seen with the highest concentration of calpain (0.08 units) at 0 hours PI (Figure 5A, right column, top panel). p53 sensitivity to µ‐calpain degradation progressively declined at 9 hours and 48 hours PI (Figure 5A, right column). As observed with lysates not pretreated with E64d (Figure 5A, left column), exogenous µ‐calpain did not increase sub‐53–kDa fragment levels. In fact, the sub‐53–kDa fragments were less abundant in the cells pretreated with E64d (Figure 5A, right column) than in the cells not pretreated with E64d (Figure 5A, left column). This suggests that another Ca^2+^‐dependent protease was responsible for generating these fragments after HCMV infection, even though exogenous µ‐calpain could degrade both some p53 and its fragments in a concentration‐dependent manner.

To test if m‐calpain may participate in p53 cleavage (Figure 5B), the cleavage assays were repeated using purified human m‐calpain. Concentration‐dependent reductions were seen in full‐length p53 at 0, 9, and 48 PI (Figure 5B, left column). Interestingly, the addition of exogenous m‐calpain resulted in the appearance of p53(ΔCp44) at 0, 9 and 48 hours PI. At 0 hour PI, exposure to 0.02 or 0.04 units of m‐calpain produced a faint p53(ΔCp44) band, but a similar band was not observed when more (0.08 units) or less (0.01 units) m‐calpain was added (Figure 5B, left column, upper panel). p53(ΔCp44) became more abundant in cell lysates treated with m‐calpain (from 0.01 units to 0.08 units) as the course of the infection progressed (Figure 5B, left column, middle, and lower panels), which was consistent with the results of immunoblot analysis of the whole‐cell lysates (Figure 1).

E64d pretreatment of HCMV‐infected cells also resulted in remarkable degradation of full‐length p53 at 0 hour PI when the higher concentrations of m‐calpain were used (0.08 and 0.04 units) (Figure 5B, right column, upper panel). The p53 fragment bands, particularly the p53(ΔCp44) band, were also more intense (0.02 and 0.01 units; Figure 5B, right column) than those observed in the absence of E64d pretreatment (Figure 5B, left column, upper panel). Exogenous m‐calpain (from 0.01 to 0.08 units) produced an increasingly greater abundance of p53(ΔCp44) and other p53 fragments as the course of the infection progressed (Figure 5B, right column). These results show that exogenous calpains are capable of degrading p53 molecules, and m‐calpain, but not µ‐calpain, is capable of cleaving p53 to generate p53(ΔCp44) and other p53 fragments in HCMV‐infected cell lysates.

### 3.5 Subcellular distribution of p53 fragments during the progression of HCMV infection

**Figure 6:**
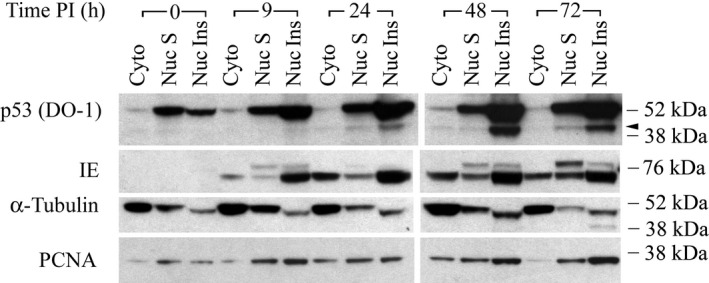
Subcellular distribution of p53 fragments during the progression of HCMV infection. Density‐arrested LU cells were HCMV‐infected (5 PFU/cell) as in Figure 1. Cells were harvested at the indicated time post infection (PI) and fractionated into a cytosolic fraction (Cyto), Buffer C‐soluble nuclear fraction (Nuc S), and Buffer C‐insoluble nuclear fraction (Nuc Ins). Polypeptides (40 μg/lane) were then analyzed by immunoblot with DO‐1 antibody. Blots were stripped and reprobed with antibody against HCMV IE protein, α‐tubulin, or PCNA. The results are representative of at least two independent biological replicates, each one in two technical replicates. Arrowhead: p53(ΔCp44). kDa sizes: the relevant molecular weight markers

The biological functions of these p53 fragments, if any, are unknown. Knowledge of their subcellular localization could provide insight in this regard. Because standard fluorescence microscopic studies using DO‐1 antibody^44^ would not be able to definitively distinguish the subcellular localization of full‐length p53 and the N‐terminal p53 fragments, HCMV‐ and mock‐infected cells were fractionated. The fractionation procedure used yielded three fractions: (a) cytosolic lysates, (b) Buffer C‐soluble nuclear extracts [see “Cell fractionation and protein extraction*”* in Experimental Procedures^55^], and (c) Buffer C‐insoluble nuclear proteins. At 0 hour after HCMV infection, p53 fragments were not evident in any of the three fractions (Figure 6). At 9 hours PI, bands corresponding to p53 fragments were detected in the Buffer C‐insoluble nuclear fraction, but not in the other two fractions. Note that the film exposure time for the findings shown in Figure 6 was not as long as that for the results shown in Figure 1. As the course of infection continued, p53(ΔCp44) became progressively more abundant in buffer C insoluble fraction, peaking at 48 hours PI, and were still substantially increased at 72 hours PI (relative to the increase at 24 hours PI). The intensities of the p53(ΔCp44) band in the cytosolic lysates were relatively much weaker. It was very faint at 24 hours and 48 hours PI, and almost undetectable at the other time pints. The intensities of the p53(ΔCp44) band in the Buffer C‐soluble nuclear extract were relatively stronger than that in the cytosolic lysates prepared at 24 to 72 hours PI, although they were still faint. Thus, p53(ΔCp44) appeared to be tightly associated with the chromatin‐rich fraction. Blots were reprobed with antibody against HCMV IE protein,^53^ which served as a reference for HCMV infection and fraction specificity. They were also reprobed with antibodies against α‐tubulin and PCNA, which served as references for cell fractionation^69^, ^70^ and cell proliferation^71^ (Figure 6).

### 3.6 Detection of polypeptides of less than 53 kDa with anti‐p53 antibody (DO‐1) in human dermal fibroblasts

**Figure 7:**
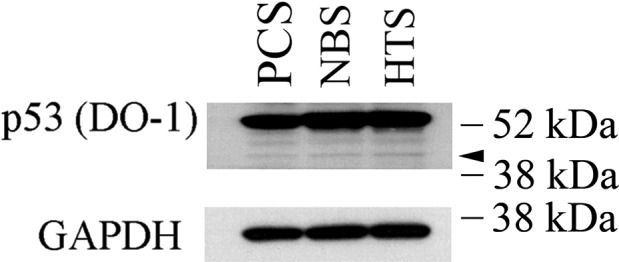
Sub‐53–kDa polypeptides recognized by p53 antibody (DO‐1) in human dermal fibroblasts. Whole‐cell lysates from three primary human skin fibroblast lines were resolved by SDS‐PAGE and probed with p53 antibody (DO‐1). GAPDH served as a loading control. The results are representative of at least two independent biological replicates, each one in two technical replicates. PCS: primary human dermal fibroblasts from normal neonatal foreskin; NBS: primary human dermal fibroblasts from non‐burn dermal tissue of a burn patient; HTS: primary human dermal fibroblasts from hypertrophic scar tissue from the same burn patient.^47^ Arrowhead: p53(ΔCp44). kDa sizes: the relevant molecular weight markers

Because endogenous human p53 N‐terminal fragments, particularly p53(ΔCp44), have not been described and because calpain activity is essential for skin wound healing and contributes to scar formation,^72^ it was of interest to determine if sub‐53–kDa fragments of p53 could be detected in human dermal fibroblasts. In addition, exposure of human fibroblasts to calpeptin, a pan‐calpain inhibitor, has been shown to reduce collagen synthesis, impair TGFβ‐induced differentiation into αSMA‐expressing myofibroblasts, and to decrease efficiency in a collagen gel contraction assay.^72^ Thus, we investigated whether p53 N‐terminal fragments were present in three primary human dermal fibroblast cell lines: PCS (human primary dermal fibroblasts from normal neonatal foreskin), human primary dermal fibroblasts from non‐hypertrophic scar skin (NBS),^47^ and human primary dermal fibroblasts from hypertrophic scar (HTS) tissue. NBS and HTS fibroblasts were from the same burn patient.^47^ All three cell lines were found to have p53 fragments that were similar to those observed in the human lung fibroblasts, ie*,* with molecular masses around ~44‐, ~47‐, and ~50‐kDa (Figure 7). These findings suggest that these fragments may be more broadly associated with cellular stress and activation of endogenous calpain.

## 4 DISCUSSION

In this study, we consistently detected p53 N‐terminal fragments in HCMV‐infected LU cells and, in much lower abundance, in mock‐infected LU cells. In particular, we observed that HCMV infection was associated with elevated levels of p53(ΔCp44). The abundance of p53(ΔCp44) increased in a biphasic manner during HCMV infection, peaking at 6 to 9 and 48 hours PI (Figure 1). Although ~47‐ and ~50‐kDa p53 fragments were also observed, these bands were rather faint and not consistently discernable. Accordingly, we focused on p53(ΔCp44). p53(ΔCp44) is unlikely to be p53β, because the abundance of p53β, as detected by the KJC8 antibody, which is specific for intron 9, did not change following HCMV infection (Figure 1). After treatment with either E64d or ZLLH, the intensity of the p53 N‐terminal fragments (~50‐, ~47‐, ~44‐kDa) detected by the p53 antibody (DO‐1) (eg, Figure 2) decreased. These and other observations (eg, Figure 4) support the view that at least some p53 in HCMV‐infected cells is susceptible to calpain‐mediated cleavage, resulting in increased levels of N‐terminal p53 fragments, particularly p53(ΔCp44), as discussed below.

The following observations indicate that p53(ΔCp44) is the product of m‐calpain cleavage of p53. (a) Treatment of HCMV‐infected cells with E64d or ZLLH, either in the presence (Figure 4) or absence of cycloheximide (Figure 3), substantially decreased the abundance of p53(ΔCp44). (b) p53 extracted from either HCMV‐ or mock‐infected cells was susceptible to cleavage by m‐calpain in vitro, which generated p53(ΔCp44) (Figure 5B); while µ‐calpain did not produce additional p53(ΔCp44) in vitro (Figure 5A), even though it digested p53. These results suggest that µ‐calpain is not responsible for generating many, if any, of the p53 fragments observed in HCMV‐infected cells. (c) The susceptibility of p53 to m‐calpain cleavage in vitro was enhanced when calpain‐sensitive p53 molecules were preserved by pretreating cells with E64d (Figure 5). (d) The increased levels of p53(ΔCp44) in HCMV‐infected cells (Figure 1) is consistent with the activation of calpain in HCMV‐, but not mock‐, infected cells, as previously reported.^45^ Since E64d not only inhibits calpain, but also inhibits cathepsin,^73^ ZLLH, a relatively specific calpain inhibitor,^74^ was used also to further examine the effect of calpain inhibition. As shown in the results (Figure 2), both E64d and ZLLH substantially reduced observed levels of p53(ΔCp44). In cells, calpain can be inhibited by calpastatin, the endogenous inhibitor of calpain. In the future work, it may be interesting to examine if over‐expression of calpastatin in LU cells reduces levels of the p53 fragments, although our previous studies revealed that the abundance of calpastatin did not change during HCMV infection.^45^

Even though calpain is activated in LU cells during HCMV infection,^45^ only a small portion of p53 molecules in HCMV‐infected cells was sensitive to calpain‐mediated cleavage (Figures 2 and 5). In fact, the cellular abundance and stability of p53 were greater in HCMV‐infected cells than in mock‐infected cells.^40^, ^41^, ^42^, ^43^, ^44^, ^75^ Given the specific reactive epitopes within the p53 molecule for each of the p53 antibodies used in this study (Table 1), our results suggest that, consistent with other reports,^40^, ^41^, ^42^, ^43^ apparent p53 levels rise during HCMV infection, as we have shown using antibodies (Bp53‐12, DO‐1, FL393) that recognize amino acid sequences near the N‐terminus of the p53 molecule^44^ or full‐length p53. A decrease in p53 was observed with Pab240, which recognizes an epitope near the middle of p53. This result may be due to post‐translational modification(s) to p53 during HCMV infection that obscure Pab240 epitopes on some p53 molecules. Although it was previously reported that the principal mechanisms underlying p53 resistance to proteasome‐mediated degradation in HCMV‐infected cells, which contribute to the increase of p53 abundance, are nuclear export and degradation of HDM2,^44^ the results of this study suggest that changes (eg, through some p53 post‐translational modification resulting in altered protein folding) in the sensitivity of p53 to calpain‐mediated cleavage may also contribute to the resistance of most p53 molecules to degradation. Other p53 post‐translational modifications may also contribute the sensitivity of some of p53 molecules to the m‐calpain‐mediated generation of p53(ΔCp44). The relationship of specific modifications of p53 to its sensitivity during HCMV infection to m‐calpain‐mediated cleavage remains to be studied.

As noted above, p53(ΔCp44) abundance underwent a biphasic increase, reaching maxima at 6 to 9 hours PI and at 48 hours PI (Figure 1). This biphasic response could result from changes in the sensitivity or availability of p53 to calpain‐mediated cleavage, as well as to alterations in the stability of p53(ΔCp44) and/or binding affinity of p53(ΔCp44) to specific antibodies during the course of HCMV infection. This biphasic pattern in p53(ΔCp44) levels is unlikely to be related to changes in calpain activity during the replicative cycle of HCMV, as calpain activity increases by more than 2‐fold at 9 hours PI, gradually intensifies to about 5‐fold (µ‐calpain) and 7‐fold (m‐calpain) relative to mock‐infected cells at 72 hours PI, and remains at these levels through at least 96 hours PI, the longest time point tested.^45^

Calpain‐catalyzed cleavage is essential to many calcium‐regulated physiological processes, such as muscle contraction, neuronal excitability, secretion, signal transduction, cell cycle progression, cell proliferation, differentiation, apoptosis, and repair of wounded cell membranes.^76^, ^77^, ^78^ Activation of calpain has been implicated in multiple pathological processes of the brain, eyes, heart, lungs, pancreas, kidneys, vascular system, and skeletal muscle, as well as in neurodegenerative disorders, cancer, and infectious diseases.^19^, ^78^, ^79^ In fact, the ubiquitous µ‐ and m‐calpains are increasingly recognized as important regulators of stress‐related responses^80^ given their potential to increase signal strength and the duration of signaling events.^18^, ^20^ Recognition of the involvement of calpains^45^ and signal transduction pathways in the pathogenesis of HCMV infection^37^, ^38^, ^39^ suggest calpain activity could be a target for therapeutic approaches for HCMV infection, as has been suggested previously for other pathologies.^78^ Because specific amino acid residues or sequences for proteolytic cleavage have not been defined for calpain‐mediated proteolytic activity,^78^, ^81^, ^82^, ^83^, ^84^ calpain‐mediated proteolysis may be associated with protein conformation. Calpains may hydrolyze substrate proteins in a limited manner, generating large fragments that retain some intact domains.^85^, ^86^, ^87^, ^88^, ^89^, ^90^ These fragments may have activities different from those of the parent protein.^91^ For example, the 18‐kDa Bax fragment generated by calpain cleavage^92^ displays a more potent ability to induce cell death than the 21‐kDa full‐length Bax.^93^ In addition, generation of a 17‐kDa neurotoxic fragment of the tau protein by calpain‐mediated cleavage may be a mechanism leading to neurodegeneration that is shared by multiple tauopathies.^94^, ^95^ Calpain‐mediated cleavage can provide a mechanism for rapidly changing the function or potency of proteins.

The N‐terminal p53 fragments were unstable (Figure 4), particularly in mock‐infected cells, and were not readily detectable in mock‐infected cells, suggesting they were degraded via ubiquitin and/or other pathways. When the cells were treated with MG132, which inhibits proteasome‐mediated protein degradation, p53(ΔCp44) was preserved in both mock‐ and HCMV‐infected cells (Figure 3), although the abundance was much greater in the latter. In HCMV‐infected cells, p53(ΔCp44) could be detected without MG132 treatment, although it was not as stable as full‐length p53 (Figure 4). By 6 or 12 hours after MG132 treatment, p53 and p53(ΔCp44) increased considerably (Figure 3). Blocking protein synthesis with cycloheximide further supported the view that MG132 treatment prolongs the half‐life of the p53 fragments, showing that the half‐life was extended to at least 24 hours, the longest time tested (Figure 4). These findings strongly suggest that p53(ΔCp44) is subject to degradation by the proteasome pathway. In full‐length p53, the ubiquitin peptide is conjugated to Lys residues located at the C‐terminus, particularly K370, K372, K373, K381, K382, and K386.^96^ Given that that C‐terminus is absent from the N‐terminal fragments generated by calpain cleavage, impaired proteasome degradation of these p53 fragments would not be surprising. Even so, p53(ΔCp44) was still subject to proteasome degradation. This paradoxical phenomenon could be due to other Lys residues present in the p53 N‐terminal fragments. Other possible mechanisms, such as indirect stabilization of p53 fragments by MG132 (eg, preserving one or more proteins, which in turn inhibit one or more degrading proteases), remain to be studied. The low abundance of p53 fragments in mock‐infected cells relative to HCMV‐infected cells may be attributable, in part, to less p53 abundance, lower calpain activity, lower stability of the p53 fragment, and a non‐compromised proteasome pathway. On the other hand, the relatively greater abundance of p53(ΔCp44) in HCMV‐infected cells may attribute to greater p53 abundance, increased calpain activity and consequently greater p53 cleavage, and extended fragment half‐life due to the compromised proteasome pathway in HCMV‐infected cells.^44^

The biological functions of the p53 N‐terminal fragments remain to be studied. Human p53 comprises 393 amino acid residues and six modular domains^13^, ^14^: (a) The N‐terminus transcription activation domain (TAD) contains two complementary transcriptional activation domains, with the major one at residues 1‐42 and the minor one at residues 55‐75, and it is specifically involved in the regulation of several pro‐apoptotic genes. (b) The proline‐rich domain (PR, residues 61‐92). (c) The central DNA‐binding core domain (DBD, residues 94‐292).^1^ (d) An oligomerization domain (OD, residues 326‐353).^1^, ^13^ (e) A nuclear localization signaling domain (residues 316‐325). (f) A C‐terminal domain (CTD), which is involved in regulation of DNA binding, p53 protein stability, and transcription cofactor recruitment (residues 364‐393).^1^, ^97^, ^98^ Among the p53 fragments that we observed by SDS‐PAGE, the fragments with a molecular mass of about 44‐kDa, 47‐kDa, and 50‐kDa could contain intact N‐terminal structures, as they were detected with DO‐1 and Bp53‐12 (see Table 1). Although p53 appears to be a 53‐kDa protein as determined by SDS‐PAGE, size calculation based on amino acid residues yields a mass of only 43.7 kDa.^99^ This difference may be attributable to the high number of Pro residues, which may slow p53 migration during SDS‐PAGE and make it appear heavier than it actually is. The molecular mass of Pro‐rich proteins determined by SDS‐PAGE may be different from that determined using other methods such as sedimentation equilibrium and gel filtration.^100^ Because the PR domain is located in residues 64‐92, the N‐terminal fragments observed should possess an intact PR domain. Accordingly, based on the electrophoretic behavior of p53(ΔCp44) in SDS‐polyacrylamide gels and considering the effect of the PR domain, p53(ΔCp44) may lack approximately 70 amino acid residues at the C‐terminus of p53. These missing residues contain the important OD and CTD, which are subject to extensive post‐translational modification, such as phosphorylation, acetylation, ubiquitination, sumoylation, methylation, and neddylation. Accordingly, this region is critical for regulation of many p53 biological functions.^1^ Nevertheless, the p53 protein has numerous other important active sites such as TAD, PR, and DBD, and many of these will be preserved in p53(ΔCp44).^13^, ^14^ TAD, for example, interacts with many proteins, such as CBP/p300, CSN5/Jab1, Mdm2, RPA, TFIID, and TFIIH.^8^

p53(ΔCp44) appears to be predominately located in the nuclei of HCMV‐infected cells in these studies and appears to be tightly associated with a chromatin‐rich fraction (see Figure 6), because it was difficult to extract with Buffer C, but could be extracted using the much stronger SUMO‐1–modified protein extraction buffer.^56^ Protein extractability may depend on the strength of detergents and/or denaturing reagents in the lysis buffers^51^, ^101^ and intermolecular protein interactions, which are associated with post‐translational modification. When the Buffer C‐insoluble pellets were carefully resuspended in SUMO‐1–modified protein extraction buffer, which contains a much higher concentration of SDS (1.275%), they became so sticky that no new pellet could be obtained after centrifugation. The fraction of Buffer C‐insoluble nuclear proteins extracted with SUMO‐1–modified protein extraction buffer may contain chromatin‐rich DNA‐binding proteins, as the formation of highly viscous nuclear samples after the addition of ionic detergents is thought to reflect the presence of high‐molecular‐weight DNA.^57^ Moreover, brief sonication to break high‐molecular‐weight DNA can reduce the viscosity, following DNA unfolding and solubilization of the nuclear proteins with a higher concentration of SDS. Nuclear localization and nuclear export of p53 can be regulated by the nuclear localization signaling domain (residues 316‐325) and the Leu‐rich nuclear export signal motif (residues 340‐351),^14^ although how N‐terminal fragments that may lack these domains enter and/or remain in nuclei will require further study. If the supposition that p53(ΔCp44) lacks about 70 amino acids at the C‐terminus is correct, p53(ΔCp44) may lack the site that is acetylated by the co‐activator p300.^11^ Acetylation of p53 can dramatically stimulate its sequence‐specific DNA‐binding activity, possibly as a result of an acetylation‐induced conformational change. Since p53(ΔCp44) also lacks the OD, it is unlikely that this species can form a homotetramer, which is critical for wild‐type p53 cellular DNA binding and is essential for the activity of wild‐type p53 in vivo.^14^ The Leu‐rich nuclear export signal motif^102^ is masked in tetrameric p53; this motif is buried within the tetramer interface and becomes exposed only after dissociation of the tetramer. Given that p53(ΔCp44) lacks nuclear localization and OD domains, the presence of this fragment in the nucleus is puzzling. One hypothesis explaining the nuclear localization of p53(ΔCp44) is that these fragments are generated from full‐length p53 that is already present in the nucleus and remain in the nucleus because they lack an export signal domain. However, this hypothesis does not explain the close association of p53(ΔCp44) with DNA binding proteins, which may involve other mechanisms. The results of this study suggest that most p53(ΔCp44) binds to DNA binding proteins or DNA (or is already bound to DNA binding proteins or DNA prior to calpain‐mediated cleavage) with very high affinity and are therefore located in the nucleus. Also, the observed nuclear localization and DNA binding affinity may be due, in part, to the DBD in p53(ΔCp44).^14^ Whether these p53 fragments bind to DNA indirectly by protein‐protein interactions and/or directly via one or more of the domains remaining in the fragments are yet to be determined. Additional studies will be needed to define the precise mechanisms underlying the nuclear localization and tight chromatin‐rich association of the p53 fragment identified here.

p53 is critical for HCMV infection and enhances the ability of HCMV to replicate. In cells lacking p53, HCMV infection is compromised and HCMV DNA accumulation is delayed and decreased.^103^ Partial reconstitution of p53 (‐/‐) cells with a wild‐type copy of p53 helps return all parameters toward the wild‐type condition, while reconstitution with mutant p53 does not.^103^ p53 is crucial for HCMV immediate early and early gene expression.^104^ The absence of p53 during HCMV infection leads to decreased UL53 expression, disrupting UL50 localization to the inner nuclear membrane and in this way, inhibits nucleocapsid nuclear egress.^105^ The cellular level of p53 is substantially increased in productive HCMV infection^40^, ^41^, ^42^, ^43^, ^44^; however, cells still enter and traverse the cell cycle to a point at or near the G_1_/S boundary,^39^ suggesting that at least some p53 functions are inactivated. The HCMV proteins IE1,^67^ IE2,^63^, ^64^, ^65^ UL44,^68^ and UL84^63^ can interact with p53, thereby altering p53‐mediated transcription. HCMV IE2 downregulates p53‐dependent gene activation by inhibiting local histone acetylation mediated by p300/CBP.^66^ It is possible that one or more of the p53 N‐terminal fragments reported here binds to p53 response elements^14^ and competes with wild‐type p53 function.

Taken together, the current results show that during productive HCMV infection a portion of p53 molecules undergo calpain‐mediated cleavage, yielding N‐terminal fragments that appear to be tightly associated with the chromatin‐rich fraction, as seen by their resistance to extraction. In vitro calpain cleavage studies suggest that m‐calpain is largely responsible for the generation of p53 N‐terminal fragments, particularly p53(ΔCp44), in HCMV‐infected LU cells. Because p53(ΔCp44) is abundant at points in the HCMV replication cycle in which the virus is modifying the cell to support HCMV proliferation, p53(ΔCp44) may have some role in the cellular pathogenesis of HCMV, [eg, cell cycle deregulation,^37^, ^38^, ^39^ susceptibility to gene mutation,^106^, ^107^ chromosome aberration,^108^, ^109^, ^110^ oncogenic transformation,^111^, ^112^, ^113^ and/or HCMV replication]. The biological activities of p53(ΔCp44) during HCMV infection remain to be studied, particularly because the p53 C‐terminal sequences reportedly participate in virtually every aspect of p53 function as a transcription factor, including DNA binding, cofactor recruitment, and protein stabilization.^1^ Understanding how p53(ΔCp44) may participate in HCMV pathogenesis is of interest, as it addresses this and other enigmas, possibly shedding light on currently unrecognized aspects of p53 regulation and function. Moreover, the finding that the N‐terminal p53 fragments were present in human dermal fibroblasts, including fibroblasts isolated from post‐burn hypertrophic scar, hints at a wider role for this p53 fragment in other cellular systems. That is, this p53 fragment may also be part of a stress‐associated, calpain‐mediated response, making it worthy of future investigation.

## CONFLICT OF INTEREST

The authors declare that they have no conflict of interest.

## AUTHOR CONTRIBUTIONS

Z. Chen and T. Albrecht designed experiments, interpreted and analyzed data, and drafted the manuscript. Z. Chen conducted all the experiments. P. J. Boor, C. C. Finnerty, and D. N. Herndon contributed to the design of experiments, interpreted and analyzed the data, and helped write the manuscript.
